# The Influence of Fatty Acids on the GpA Dimer Interface by Coarse-Grained Molecular Dynamics Simulation

**DOI:** 10.3390/ijms150814247

**Published:** 2014-08-15

**Authors:** Nadine Flinner, Oliver Mirus, Enrico Schleiff

**Affiliations:** Cluster of Excellence Macromolecular Complexes, Center of Membrane Proteomics, Department of Biosciences, Molecular Cell Biology of Plants, GU Frankfurt am Main, 60439 Frankfurt, Germany; E-Mails: nadine-flinner@gmx.de (N.F.); o.mirus@bio.uni-frankfurt.de (O.M.)

**Keywords:** Glycophorin A dimerization, dimer interface formation, bitopic transmembrane α-helix, fatty acid dependency, MARTINI force field

## Abstract

The hydrophobic thickness of membranes, which is manly defined by fatty acids, influences the packing of transmembrane domains of proteins and thus can modulate the activity of these proteins. We analyzed the dynamics of the dimerization of Glycophorin A (GpA) by molecular dynamics simulations to describe the fatty acid dependence of the transmembrane region assembly. GpA represents a well-established model for dimerization of single transmembrane helices containing a GxxxG motif *in vitro* and *in silico.* We performed simulations of the dynamics of the NMR-derived dimer as well as self-assembly simulations of monomers in membranes composed of different fatty acid chains and monitored the formed interfaces and their transitions. The observed dimeric interfaces, which also include the one known from NMR, are highly dynamic and converted into each other. The frequency of interface formation and the preferred transitions between interfaces similar to the interface observed by NMR analysis strongly depend on the fatty acid used to build the membrane. Molecular dynamic simulations after adaptation of the helix topology parameters to better represent NMR derived structures of single transmembrane helices yielded an enhanced occurrence of the interface determined by NMR in molecular dynamics simulations. Taken together we give insights into the influence of fatty acids and helix conformation on the dynamics of the transmembrane domain of GpA.

## 1. Introduction

All living cells are surrounded by membranes, which are mainly composed of glycerophospholipids, sphingolipids and sterols [1,2], which consist of a hydrophilic head group and a hydrophobic tail. The lipids are arranged in two layers within the membrane, with the tails packed against each other. Glycerophospholipids are the most prominent components of biological membranes and phosphatidylcholine (PC) is the most frequently occurring lipid in biological systems [3]. 

By molecular dynamics (MD) simulations it has been shown that PC lipids with saturated and unsaturated fatty acid chains separate spontaneously into an liquid ordered phase, containing the saturated lipids and cholesterol, and an liquid disordered phase, containing the lipids with unsaturated fatty acids [4,5]. A small mismatch between the saturated and unsaturated fatty acids reduces the driving force for segregation, while the increase of cholesterol leads to an enhanced driving force for the phase separation [6]. Phase separation and compartmentalization are also present in biological membranes, for example lipid rafts are observed in the eukaryotic plasma membrane, which are enriched in sphingolipids and cholesterol [7].

Native lipid bilayers are generally crowded with transmembrane proteins [7,8,9], which influence and which are influenced by the biophysical properties of the membrane. By MD simulations it has been shown that bitopic α-helices are sorted into the liquid disordered phase [6]. This results from the enhanced number of possible van der Waals contacts [10], independently of the hydrophobic thickness of the different phases [11]. However, some proteins, e.g., peripheral membrane proteins H-Ras or Hedgehog are sorted into the liquid ordered phase, which depends on their lipid anchor [12].

Irrespective of whether the transmembrane region is sorted into the liquid disordered or ordered phase, it is generally observed that the functional activity of membrane proteins depends, among other things, on the hydrophobic thickness and the composition of the membrane [13]. The highest activity of proteins is often observed in case that no hydrophobic mismatch between the protein and the membrane occurs [14]. To compensate for the hydrophobic mismatch, α-helical transmembrane regions can change their tilt angle, the conformation of side chains or their effective hydrophobic length [15]. Alternatively, the membrane can adapt its thickness or can undergo a midplane bending to scope with the transmembrane domain dimension [10,13]. To minimize the energetic penalty for this mismatch, membrane proteins often aggregate after occurrence of a hydrophobic mismatch [16]. Consequently, changing the fatty acid chain length would have significant effects on the packing of the α-helices [10].

Glycophorin A (GpA) is one of the model peptides used to investigate the biophysical properties of membrane domain behavior in lipid environment. GpA is a human protein of 150 amino acids with a single transmembrane helix and is localized in the plasma membrane of red blood cells. This membrane consists of phospholipids, cholesterol and glycolipids. Among the phospholipids PC, phosphatidylethanolamine, and phosphatidylserine are most abundant and the fatty acids thereof have a length of 16 to 22 carbon atoms [17,18]. GpA carries an *O*- and *N*-glycan, which again carries sialic acid. This negative charge prevents aggregation of the red blood cells and enables the flow through the blood vessels [19]. To exhibit its function, GpA forms a homodimer as well as a heterodimer with Glycophorin B, which then forms a multimeric protein complex with other proteins like band 3 [18,20]. The structure of the homodimerizing membrane inserted segment of GpA in micelles [21,22] as well as in bicelles [22] was determined by NMR spectroscopy. The interface between the two helices consists of a GxxxG motif, a common motif for helix-helix association [23]. GpA forms a right-handed dimer with a crossing angle of approximately −40° [21]. The interface does not contain hydrogen bonds between the two monomers, while the OH group of threonine localized in the dimerization interface forms an intramolecular hydrogen bond with the backbone [21].

Although GpA dimerization has been studied in silico by MD simulations (e.g., [24,25,26]), the dependence of the dimeric interface on fatty acid chain length has not yet been investigated in detail. Thus, we examined the influence of different fatty acid chain lengths and saturation degrees on the stability and dynamics of the GpA homodimer by coarse-grained molecular dynamics (CG-MD) simulations with the MARTINI force field [27,28,29]. We performed self-assembly simulations with the transmembrane region of GpA and analyzed whether initial interface formed after assembly depends on the fatty acid composition of the membrane. Subsequently, we analyzed the fatty acid dependence of the transitions between different dimer interfaces as well. Finally, simulations on the stability of the dimer observed by NMR have been performed to explore the importance of the correct helix topology on the stability.

## 2. Results

### 2.1. Self-Assembly of the GpA-Transmembrane Region Reconstructs the NMR Interface with Low Frequency

We used the transmembrane part of GpA and performed CG-MD simulations in five different membranes to investigate the influence of the fatty acids on interface formation of the dimeric α-helical protein GpA. Each membrane consisted of lipids with different fatty acid chains, while the PC-head group was kept, because it is the most frequent head group in the plasma membrane [3], the natural environment of GpA [18,20]. We used the saturated lipids DPPC (dipalmitoyl-PC) and DSPC (distearoyl-PC) with different length, where each fatty acid is modeled by four and five CG beads representing saturated fatty acids of 15–18 or 18–21 carbons respectively. Further, a lipid with one saturated and one monounsaturated fatty acid chain (POPC: palmitoyl-oleoyl-PC) is used, with the saturated fatty acid modeled by four and the unsaturated one by five CG beads. In addition, membranes composed of the unsaturated DOPC (dioleoyl-PC with both fatty acids modeled by five CG beads) and the di-unsaturated DUPC (dilinoleyl-PC with both fatty acids modeled by four CG beads) have been included in our analysis. The hydrophobic thickness of the according membranes ranges from 2.49 ± 0.04 nm for DUPC to 3.86 ± 0.04 nm for DSPC (DUPC < DPPC (3.13 ± 0.09 nm) < POPC (3.22 ± 0.03 nm) < DOPC (3.42 ± 0.02 nm) < DSPC).

In each of the created lipid environments 300 self-assembly simulations for GpA were performed using the Martini 2.2 force field [29]. The individual simulation was terminated after formation of a stable dimer (methods) of the transmembrane regions. At first the crossing angles of the dimers was analyzed yielding mainly negative crossing angles characteristic for right-handed dimers in the thin membranes, while in thicker membranes crossing angles around 0° have been observed as well. In contrast, positive crossing angles characteristic for left-handed dimers were hardly observed in any simulation. Consistently, the crossing angle observed by NMR analysis is −40° [21]. 

**Figure 1:**
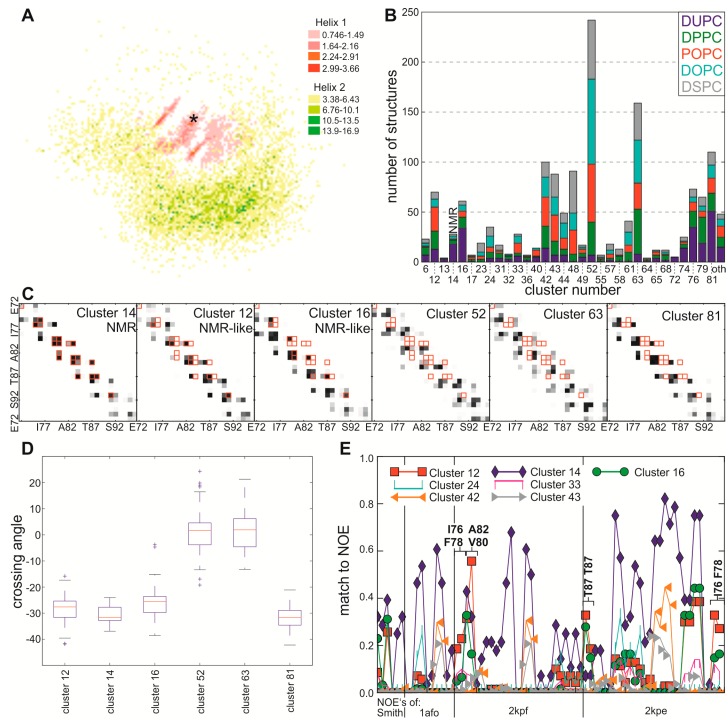
Short self-assembly simulations (25 mer) (**A**) Density map of all self-assembled dimers in top view, superimposed on BB beads of helix A. The “*” marks the position of T87 of the first helix; (**B**) The frequency of the different interfaces obtained for each lipid (DUPC…blue, DPPC…green, POPC…red, DOPC…cyan, DSPC…grey) after UPGMA clustering is shown in total numbers. Only clusters containing at least three structures of different frames (0.1%) are shown, all structures from other clusters are shown in the category “oth.” (others); (**C**) Shown are residue-residue contact maps for all structures in the corresponding cluster. The frequency of contacts is shown as grey scale with black indicating contact present in all structures of the cluster and white indicating contact not present. Framed in red are contacts observed by NMR analysis [21]. For representative CG-structures please see Figure S1; (**D**) The box plot shows the distribution of the crossing angles of the structures in each of the clusters shown in **B**. A box contains 50% of the data values and marks the 75th percentile and the 25th percentile, whereas the red line marks the median. The whiskers (horizontal lines) mark the values, which lie within the 1.5 interquartile range, which is defined as the box length; further values are marked as outliers (“+”); (**E**) Agreement of the backmapped structures with interhelical NOE constraints from NMR experiments. On the *X*-axis the single NOE constraints from four experiments (Smith51: micelle or POPC; 1afo [21]: micelle; 2kpf [22]: bicelle; 2kpe [22]: micelle) and on the *Y*-axis the percentage of structures in the corresponding cluster which fulfill this distance are plotted. Shown are only structures with a frequency at least as high as the NMR cluster.

In general, most of the self-assembled interfaces are located at one side of the helix. Surprisingly the known dimerization interface, containing the amino acid T87, is located on the opposite side of these frequently obtained structures (Figure 1A). Next, we clustered the structures based on their interface contact plots (methods). We used all structures from self-assembly simulations mentioned in this manuscript to determine the “cluster space” and to assign identical cluster numbers to interfaces obtained with different approaches. In total 83 clusters of different interface architectures are observed.

The interfaces most frequently observed considering all short self-assemblies simulations are represented by cluster 52, which at the same time is the most frequently observed cluster for POPC, DOPC and DSPC (Figure 1B). The dimerization interface of the in cluster 52 is located at a different site than the interface of the NMR structure (Figure 1C) and has an average tilt angle near zero (−0.95° ± 6.58°; Figure 1D). The most frequent interface observed in the DPPC bilayer is represented by cluster 63, which is at the same time the second most frequently observed cluster for all short self-assemblies simulations (Figure 1B,C). The crossing angle of the dimer of the structures in this cluster is 1.17° ± 6.92° (Figure 1D). The most frequently occurring interface in the DUPC bilayer is cluster 81 (in total the third most frequently observed cluster; Figure 1B,C), which contains an asymmetric right-handed dimer with a crossing angle of −32.05° ± 4.18° (Figure 1D).

The structures forming the interface observed by NMR analysis [21] are represented by cluster 14, which has been observed for all membranes analyzed. The dimeric structures therein are right-handed with an average crossing angle of −30.81° ± 3.46°, which is slightly smaller when compared to the NMR structure (Figure 1D). Interestingly, although the interface determined by NMR analysis is generally detected with low frequency, it is most frequent in the bilayer with the smallest hydrophobic thickness (DUPC; Figure 1B).

To determine whether another interface would also explain the distances obtained by NMR experiments, the coarse grain structures from the short self-assembly simulations were backmapped into a full atomistic resolution. Subsequently we checked which of the NOE distances are fulfilled by the structures of each cluster (Figure 1E). As expected, the structures of cluster 14 match best to the NOE distances with on average 19.7 constraints fulfilled by one structure, while other clusters match some of the NOE’s even better than the NMR structure. For example, there are seven NOE constraints of GpA measured in bicelles or micelles [22] which are fulfilled most often by the structures from cluster 12 (Figure 1E). This cluster represents an interface with a crossing angle of −28.56° ± 5.50° (Figure 1D) and which contains contacts for the GxxxGxxxT motif similar to that found by NMR, but which is slightly asymmetric (Figure 1C). These seven NOE constraints are also fulfilled by structures of clusters 16 (crossing angle: −26.06° ± 6.45°) and 33 (crossing angle: 29.38° ± 9.68°) (Figure 1E), where at least the glycine residues of the GxxxG motive have contacts with each other (Figure 1C). However, the crossing angle of cluster 33 is not comparable to the one observed by NMR analysis. Thus, clusters 12 and 16 are annotated as “NMR-like” and represent together with the NMR cluster 14 about 10.6% of all self-assembled structures, and thus still not a large portion of all observed interfaces.

### 2.2. Interface Frequencies Are Depend on Different Fatty Acid Chain Environments

Next, the dependency of the preferred interface on the fatty acid chains was analyzed in more detail. At first, the probability for the occurrence of a novel interface after a defined number of simulations performed was determined. This analysis revealed a probability of 5% to find a new interface after 150 simulations, which in turn accounts for a 95% confidence for complete sampling. Furthermore, a 98% confidence is reached for the detection of all possible structures after 300 simulations. Interestingly, interfaces that occur only after 150 simulations are very unstable and short living, and most likely represent intermediates between two stable interfaces. This in turn indicates that we have sampled nearly the whole interface space.

**Figure 2:**
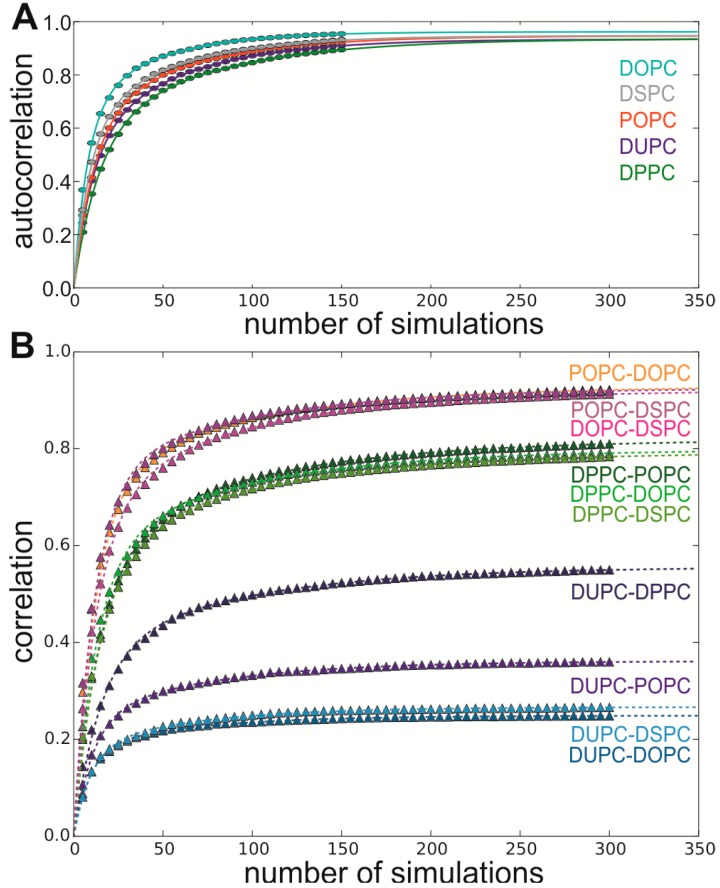
(**A**) Correlation and (**B**) Autocorrelation for the short self-assembly simulations were calculated using Pearson’s correlation coefficient (*Y*-axis) between the cluster distributions obtained from n simulations (*X*-axis). Plotted are the average values obtained from 1000 repetitions (sampling without replacement).

Further we investigated, whether we have sampled enough to determine differences in the cluster frequencies observed in the different lipids. We calculated the autocorrelation for the function of cluster frequencies using Pearson’s correlation coefficient (*r*) for *n* (*n* = 5 to 150) randomly selected interfaces of our 300 simulations without replacement and fitted a double exponential function to our data (Figure 2A). In general, correlation and autocorrelation functions decay or rise with several numbers of exponential terms, related to the number of underlying processes [30,31]. In our case, the first term refers to the number of simulations to detect all important clusters and the second term refers to the number of simulations to cover the distribution. The limits of the fitted functions are in the range of ~0.95 (Table 1). With *n* = 150 simulations r is already in the range of 0.93 (DPPC) to 0.96 (DOPC), reflecting that we have a high overlap for two distinct sets and that the sampling is nearly fully completed (Figure 2A). Thus, consistent with the probabilities to detect a new interface, we conclude that the sampling nearly covers the underlying probability distribution of the different clusters for all lipids.

**Table 1:** Optimized values for *a*, *b*, *c*, *d* and the limit of the fitted (auto-)correlation function.

Lipid	*a*	*b*	*c*	*d*	Lim
DUPC-DUPC	0.51	0.10	0.42	0.02	0.93
DUPC-DPPC	0.37	0.07	0.18	0.01	0.56
DUPC-POPC	0.24	0.09	0.12	0.01	0.36
DUPC-DOPC	0.18	0.11	0.07	0.02	0.25
DUPC-DSCP	0.18	0.10	0.09	0.02	0.27
DPPC-DPPC	0.55	0.07	0.38	0.01	0.94
DPPC-POPC	0.58	0.07	0.24	0.01	0.82
DPPC-DOPC	0.58	0.08	0.22	0.01	0.80
DPPC-DSPC	0.57	0.07	0.22	0.01	0.79
POPC-POPC	0.59	0.09	0.36	0.02	0.95
POPC-DOPC	0.73	0.08	0.20	0.01	0.93
POCP-DSPC	0.67	0.08	0.25	0.01	0.92
DOPC-DOPC	0.65	0.13	0.31	0.02	0.96
DOPC-DSPC	0.73	0.09	0.19	0.01	0.93
DSPC-DSPC	0.59	0.11	0.35	0.02	0.95

Finally, we calculated the correlation between different lipids, again using Pearson’s correlation coefficient for *n* (*n* = 5 to 300) interfaces (Figure 2B). The correlation between the three longest lipids (POPC, DOPC and DSPC) is very similar to the autocorrelation. This indicates that there is a large overlap between the distributions of the different interface clusters found for these lipids. The comparison of DUPC (shortest fatty acid chain analyzed) with POPC, DOPC and DSPC shows correlation values between 0.27 and 0.36, whereas the correlation of DPPC (longer than DUPC but shorter than POPC, DOPC and DSPC) in comparison to POPC, DOPC and DSPC is much higher with a value of about 0.80. The value for the correlation between DUPC and DPPC is intermediate with 0.56.

### 2.3. Transitions between Dimer Conformations Are Visited by Long Self-Assembly Simulations

The results presented above suggest that the preferred interface of GpA depends on the nature of the fatty acid used for membrane construction. However, the interface found by NMR experiments is observed only with low frequency in self-assembly simulations, irrespective which fatty acid is used. This prompted the question whether the interfaces observed are only transition states to a putative final interface. To this end we conducted long self-assembly simulations (10 µs) with focus on bilayers composed of DUPC (10 simulations), DPPC (10 simulations) and POPC (20 simulations). DOPC and DSPC containing membranes were not analyzed because the interface distribution observed in these membranes is comparable to the one in POPC (Figure 2). Additional, the long simulations enabled us to analyze the transitions between interfaces.

**Figure 3:**
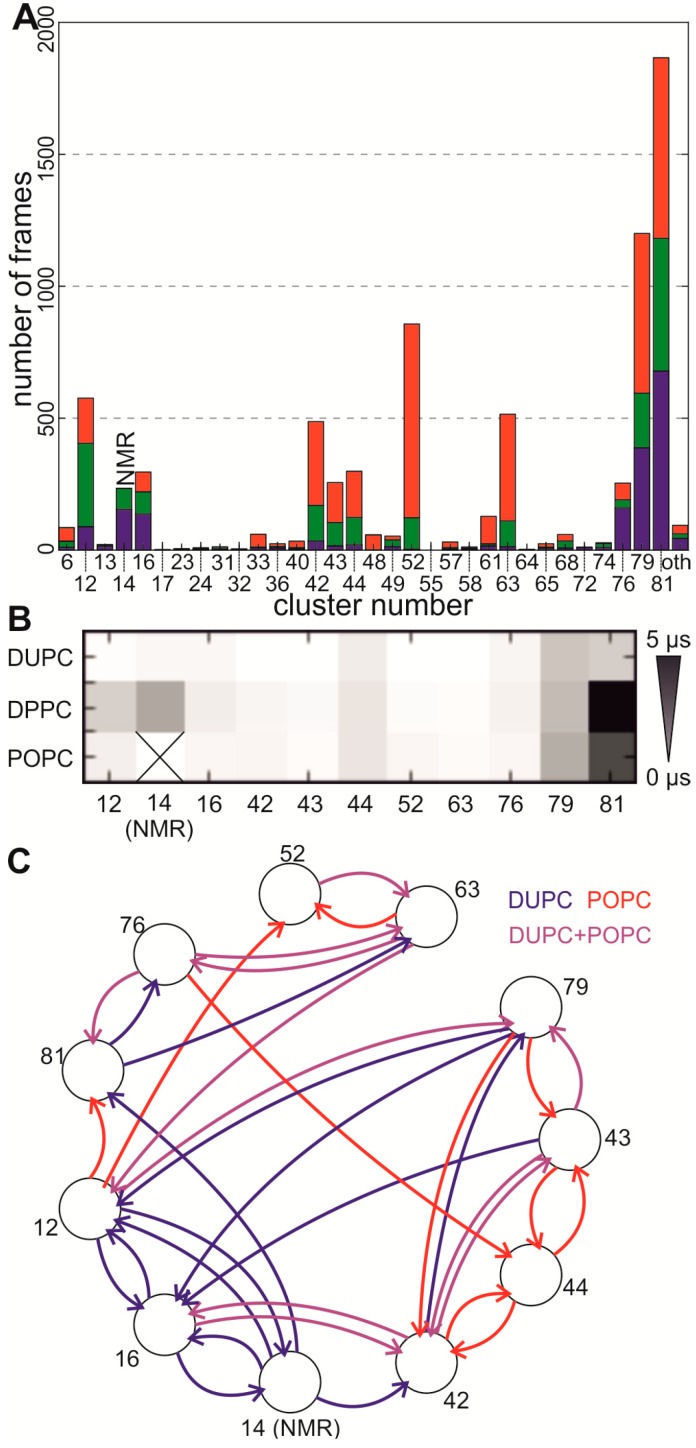
Long self-assembly simulations (25mer) (**A**) Frequency of the different interfaces obtained in the long self-assembly simulations (10 µs) with the 25 mer for each lipid (DUPC…blue, DPPC…green, POPC…red) after UPGMA clustering. Shown are the same clusters as in Figure 1B; (**B**) Retention times of different clusters in different lipids; (**C**) Transition map for the self-assembly simulations in DUPD and POPC. Each cluster with a frequency at least equal to the cluster of the NMR structure is represented by a circle, please note that the NMR cluster (12) is not found in POPC. Only the three most frequent transitions for each cluster are shown, where transitions occurring only once were excluded. Transitions occurring frequently only in DUPC were colored in blue, only in POPC in red and in DUPC and POPC in purple.

In general, all clusters observed with a frequency of at least 3% in the short self-assembly simulation of GpA in a membrane composed of one specific lipid were also observed in the long self-assembly simulation in the according membrane (compare Figure 1B with Figure 3A). This indicates that we have generally sampled the same interface space with the short and long simulations. For DUPC and POPC even all clusters with a frequency of at least 1% were visited, indicating that the sampling is more complete in membranes composed of these two lipids. However, we observed a shift of some cluster frequencies in comparison to the short self-assembly, which is caused by a high retention time e.g., in cluster 79 or 81 (Figure 3B).

Similar to the results for the short self-assembly simulations, we observe differences in the cluster frequencies of different fatty acid chains. For example, the interface of cluster 52 is more frequent in POPC and, remarkably, the cluster, which contains the contacts of the NMR structure (cluster 14), occurs only in DUPC and DPPC (Figure 3A). Interestingly, all frequently occurring interfaces convert into each other—at least via some intermediate steps—meaning that the underlying graph is strongly connected.

In general, the transition maps for DPPC and POPC are similar (Figure S2) and all transitions which were frequently observed in DPPC are also present in POPC, but due to the limited sampling and higher retention times we have less confirmed transitions for DPPC. The transitions of DUPC and POPC are in general similar as well, but a significant difference with respect to the outgoing transitions from NMR-like clusters 12 and 16 exists (Figure 3C). In DUPC the most frequent transition of cluster 12 and 16 points to the NMR interface, whereas in POPC this transition is not observed. Similarly, the second or third most transition points to the other NMR-like cluster, which is also not observed in POPC. Hence, the NMR-like interfaces are observed while simulating the dimer in both lipids, while the transition to the NMR interface is only observed in DUPC. Thus, we do not only see an influence of the fatty acid chain onto the frequency of different interfaces, we also see an influence of the preferred transitions between different clusters.

### 2.4. Stability of the NMR Interface Depends on Helix Length and Helix Conformation

**Figure 4:**
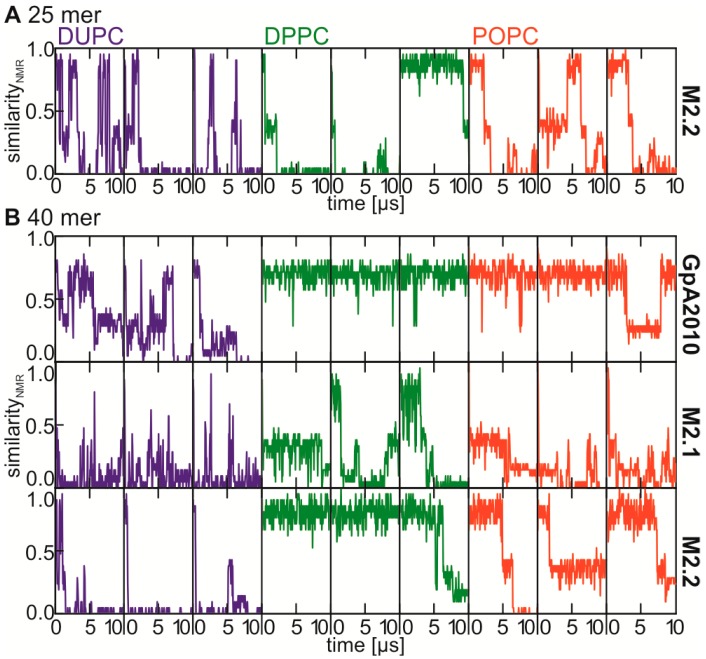
The similarity (similarityNMR) of the interfaces of the structures from trajectories obtained with different lipids (DUPC…blue, DPPC…green, POPC…red) and (**A**) the 25 mer (**B**) the 40 mer and different force field versions (GpA2010…first panel, Martini2.1…second panel, Martini2.2…third panel) to the one determined by NMR spectroscopy in micelles (1afo, [21]) is plotted against the simulation time (time). For each lipid + force field combination three simulations started with different seeds are shown. A value of one indicates that all residue-residue contacts present in the NMR structure are reproduced, while zero indicates no reproduced contact.

Next, we aimed to explore the reason for the underrepresentation of the NMR interface in our simulations. At first we tested the stability of the NMR dimer in the force field used. We embedded the structure determined by NMR analysis [21] truncated to the 25 mer into membranes of DUPC, DPPC and POPC and simulated each system three times using different seeds. We realized that the NMR interface is not stable for 10 μs in any of the simulations. Interestingly, the interface changes rapidly in DUPC, however the NMR interface reforms in all three repetitions (Figure 4A). Further, it appears that the stability of the dimer interface increases in thicker bilayers and is more stable in bilayers composed of saturated fatty acid chains (DPPC) in comparison to bilayers containing unsaturated fatty acid chains (DUPC, POPC, Figure 4A). We repeated the simulation with the construct used for NMR analysis (40 amino acids) to test if the interface is stabilized by flanking residues. In addition, indeed we observed a stabilization of the NMR interface (Figure 4A *vs.* Figure 4B). However, as found for the 25 mer, the stability of the interface of the 40mer increase in saturated or thicker membranes (Figure 4B).

In parallel, we used the MARTINI force field version Martini2.1 [28] and GpA2010 [26], because a successful reconstruction of the NMR interface was reported while using GpA2010. For GpA2010 in five of nine simulations the interface is maintained over 10 μs, while the same was observed for only two of nine simulations using the Martini 2.2 version (Figure 4B). In contrast, the Martini 2.1 version is not able to stabilize the interface at all, and in most cases the NMR interface is lost during the first microsecond (Figure 4B). However, the comparable behavior in maintaining the NMR interface while using the GpA2010 and Martini 2.2 force field is not reflected by the force field parameters. In general, Martini 2.1 and Martini 2.2 use by and large the same angles, dihedrals and bead types to model the properties of the side chain and the backbone [28,29]. In contrast, GpA2010 uses different bead types to describe non-bonded interactions and also the definition of the conformation of α-helix differs drastic as GpA2010 places the backbone beads to the CA position and not the center of mass of the amino acid backbone. Through this change different bond length and bond angles are needed. Furthermore, the secondary structure is maintained by dihedral angles in Martini 2.1/Martini 2.2, while in GpA2010 an additional bond is placed between backbone beads *i* and *i* + 4. However, one major difference between Martini 2.1 and Martini 2.2 is the bond length between the backbone beads (Martini 2.1: 0.35 nm *vs.* Martini 2.2: 0.31 nm [28,29]). This suggests that a suitable definition of the helix is at least as important as the definition of the interaction energies between different beads.

### 2.5. Correction of the Helix Conformation Leads to Increased NMR Interface Frequency

**Figure 5:**
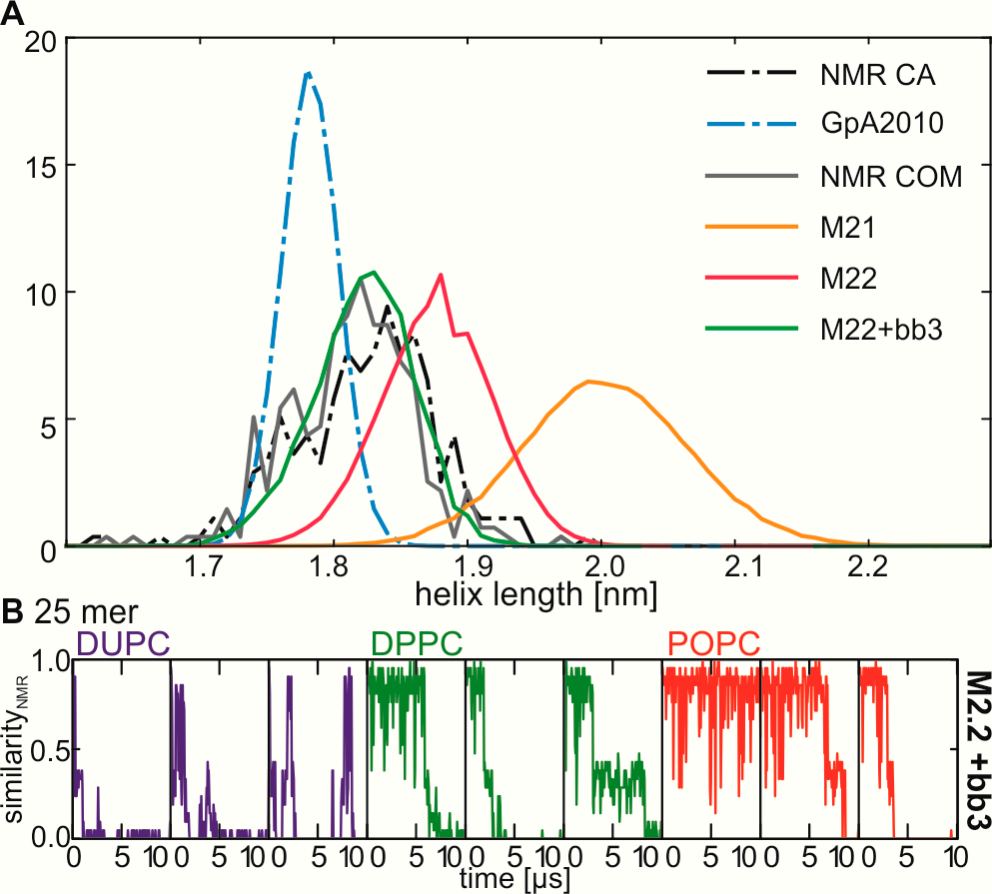
Influence of the helix conformation onto the simulation. (**A**) probability distribution for the helix length obtained from solved structures and from simulations using the COM position (solid lines) or the Cα position (dashed lines). The helix length is measured between backbone atom *i* and *i* + 12. Black/grey…NMR reference, red…M2.2, orange…M2.1, green…M2.2 plus bbi-bbi + 3 bond, blue…GpA2010; (**B**) Stability of the NMR interface of the transmembrane region using the modified M2.2 topology with additional bbi-bbi + 3 bonds. The figure is represented as described for Figure 4.

Consequently, we compared the helix length obtained from NMR experiments of different bitopic α-helices able to form dimers with the values obtained in our simulation. Consistent with earlier reports [29] we realized that the helix length within the Martini 2.1 force field is significantly longer than found by experimental approaches (Figure 5A). In turn, the experimental helix length distribution overlaps with the distribution in the other two force fields, although the length is slightly shorter (GpA2010) or longer (M2.2) in the force fields. This supports our thesis that the helix conformation is—next to the non-bonded interactions—the second determinant for a correct interface formation.

In order to test whether the helix topology has an impact on the dimerization behavior we used an elastic network-like approach similar to the normal elastic network used with the MARTINI force field [32] to model the helix topology. For this procedure we add additional bonds between the backbone atoms *i* and *i* + 3, *i* and *i* + 4 and *i* and *i* + 5 with an equilibrium length corresponding to the average observed in the known structures (0.49, 0.61 and 0.82 nm) and screen all combinations for force constants ϵ {None, 500, 1,000, 5,000, 10,000} (Table 2). We realize that adding only a bond between backbone atom *i* and *i* + 3 with a force constant of 5,000 performs best in reproducing the helix length of known structures (Figure 5B). Using this additional bond we repeat the simulations for the stability of the 25 mer NMR interface and noticed that behavior of the NMR interface of the 25 mer of GpA is not altered drastically, as a reinvestigation of the NMR interface as well as the increased stability in thicker membranes can still be observed (Figure 5B). Furthermore, the retention time of the NMR interface is slightly enhanced compared to the original Martini 2.2 force field (Figure 4A *vs.* Figure 5B).

**Table 2:** Screen for a proper force constant for backbone atoms *i* and *i* + 3/*i* + 4/*i* + 5 for GpA in DUPC. An additional screen in DPPC results in the same minimum. Shown is the difference between the probability distribution obtained from the NMR structures and the corresponding simulation.

	bb5	None	500	1000	5000	10000	bb3
bb4							
**None**		133.07	115.7	100.2	60.34	57.02	**None**
**500**		137.10	123.6	114.4	72.56	62.92	
**1000**		121.99	114.0	104.0	70.09	62.57	
**5000**		83.59	84.61	82.69	73.35	71.52	
**10,000**		75.11	77.43	76.97	76.83	79.96	
**None**		71.72	61.61	51.89	40.38	44.57	**500**
**500**		92.88	83.73	70.28	51.18	50.20	
**1000**		81.57	78.54	69.55	51.53	52.24	
**5000**		58.17	60.20	60.39	61.08	63.49	
**10,000**		64.70	66.86	67.18	73.00	76.51	
**None**		54.63	56.27	45.74	39.17	47.65	**1000**
**500**		87.97	79.35	69.90	50.15	50.48	
**1000**		75.77	72.60	65.51	51.49	52.83	
**5000**		56.98	60.49	59.77	60.89	63.18	
**10,000**		64.17	67.19	66.17	72.05	76.92	
**None**		27.38	32.15	34.26	46.53	57.14	**5000**
**500**		48.72	50.96	49.47	50.73	56.61	
**1000**		49.67	49.56	49.15	51.87	59.93	
**5000**		53.08	56.04	55.63	63.25	71.04	
**10,000**		67.34	68.75	68.40	75.04	80.79	
**None**		32.34	33.56	37.52	55.03	64.71	**10,000**
**500**		38.60	41.83	45.41	53.83	65.94	
**1000**		40.73	44.60	46.12	57.61	66.66	
**5000**		59.00	60.18	59.61	69.77	75.88	
**10,000**		71.35	71.95	73.49	79.16	84.68	

**Table 3:** Frequencies (freq) and placing for interface clusters (nr) from all short self-assembly simulations in DUPC and DPPC obtained with different force field versions (m22, GpA2010 and m22 + bb3 − m22 together with additional bond between backbone atom *i* and *i* + 3).

Place	DUPC						DPPC					
	m22		m22 + bb3		GpA2010		m22		m22 + bb3		GpA2010	
	nr	freq	nr	freq	nr	freq	nr	freq	nr	freq	nr	freq
**1**	81	17.0%	81	13.3%	2	31.3%	63	15.0%	52	19.7%	2	15.7%
**2**	76	11.7%	NMR	10.3%	0	19.3%	52	11.0%	63	12.7%	0	11.7%
**3**	16	11.3%	76	9.3%	12	12.3%	79	8.7%	42	8.0%	7	10.0%
**4**	79	6.3%	33	6.7%	16	4.7%	42	7.3%	76/79	5.0%	6	6.7%
**5**	NMR	6.0%	16/79	6.0%	6/9	3.0%	81/12	6.0%	12	4.3%	12	6.0%

These results have been promising and thus, we repeated the short self-assembly simulations to test whether the frequency of the NMR interface is increased. Indeed the frequency of NMR interface has nearly doubled in DUPC (Table 3) and resembles the second most frequent cluster. Interestingly, DUPC is the lipid which hydrophobic length comes close to the detergent fatty acid which was used for structure determination. Furthermore, using the adopted force field we still observed a fatty acid dependent shift in cluster frequencies (Table 3). This documents that the fatty acid dependency of the interface formation and stability is reproducible and not strongly dependent on the helix topology.

## 3. Discussion

The low frequency of the detection of the interface observed by NMR analysis also prompted a methodological discussion. Several technical aspects might contribute to this observation. (i) The MARTINI force fields use beads with an effective size of σ = 0.47 nm, which stands in contrast to the minimal distance between beads in different bitopic α-helices of dimers (smaller than 0.47 nm; Table S1). Furthermore, an inter-helical hydrogen bond between the threonine side chain of the first helix and the backbone of the second helix exists in membranes [33] that cannot be modeled with the current MARTINI force field. Here, the announced parameter for polarizable particles for the backbone [34] might provide a solution for future analysis; (ii) The helix conformation might change in thicker membranes, and the in here constrained helix topology adapted to values observed in detergent micelles would not be correct. However, arguing against a major impact of such phenomenon, our simulations show an increased retention time of the NMR interface in thick bilayers (Figure 4 and Figure 5) parallel FRET experiments, by which the highest dimer fraction is found in membranes with 20:1 and 22:1 fatty acids, while the observed dimer fraction is lower in thinner membranes (14:1, 16:1 and 18:1) [35]. However, the correct parameterization of bonded parameter strongly influences the dimer interface formation. There is a magnitude of different elastic network methods to restrain the overall structure of proteins like SAHBNET [36], IDEN [37] or ElNeDyn [32]. Testing all was beyond the scope of this manuscript, because we were interested in the question if different fatty acid chains influence the dimerization interface and not primarily in the explicit structures formed. Nevertheless, reproducing the helix conformation known from NMR experiments by application of an additional bond, which represents a simple elastic network with a small distance cutoff (*Rc* = 0.49 nm) and a big spring force constant (Kspring = 5000), significantly enhanced the frequency of the NMR interface, while the effects of the fatty acids are still observable (Figure 5). Thus, the major finding presented in here concerning the fatty acid dependence of dimer formation is not dependent on the correct parameterization of bonded parameter.

The fatty acid dependence of the dimerization of GpA was explored using membranes composed of different fatty acid chains with PC head group. The use of the PC head group is rationalized as (i) conclusions on fatty acid impact requires a constant head group behavior; (ii) NMR studies on GpA have been conducted in DPC micelles or DMPC/DHPC bicelles [22]; and (iii) PC is one of the most abundant head group in the plasma membrane of erythrocytes [17,18]. The analysis of the different fatty acids was prompted by the observation that the dimer of “serine zipper” peptides is more stable in thick bilayers (POPC) when compared to thin bilayers (DLPC) or micelles [38]. In line, the GpA dimer observed by NMR was better reproduced by atomistic MD simulations in a very thin bilayer (DMPC) than in a micelle [39]. The CG-MD analysis of the dynamic behavior of the GpA membrane anchor in bilayers composed of lipids with nearly physiological fatty acid chain length reproduced the interface observed by NMR in all membranes (Figure 1), but with highest frequency in membranes with a small hydrophobic thickness (DUPC, Figure 1 and Figure 3). In turn, stabilization of the dimer with characteristic negative crossing angles and NMR contacts depends on proper hydrophobic thickness of the membrane. Consequently, in thin membranes (like DUPC) no stabilization occurs and in very thick membranes (like POPC) the helices are not able to form the characteristic contacts and the negative crossing angle of the NMR interface as the tilt of the monomers is too small.

However, many different interfaces have been identified by CG-MD self-assembly analysis, which parallels the proposed large conformational space for the GpA dimer based on surface-based predictions [40]. Thus, it is tempting to speculate that the observed alternative dimeric conformations reflect components of the large structural ensemble in native membranes. Supporting this hypothesis thermodynamic measurements of single amino acid mutants of GpA indicate that the GxxxG motif is not necessary for dimerization of GpA [41]. Further, different interfaces can be adopted by the same sequence as exemplified for different bitopic α-helical proteins: For example for VEGFR-2 (vascular endothelial growth factor receptor 2) was described that an inactive and an active interface can be adopted, with helices rotated along the helical axis by 180° [42]. Similarly, EphA1 (Erythropoietin-producing hepatocellular receptor) forms a dominant right-handed dimerization interface, but a left-handed dimer interface was observed in NMR experiments as well [43]. The same two dimerization motifs were found for EphA2 and the preference for the interface depends on the hydrophobic thickness of the membrane [44]. Finally, APP (amyloid precursor protein) forms different interfaces of the transmembrane dimer as well, as identified by NMR measurements of the wild type transmembrane sequence in POPC:POPS:CHOL and POPC:POPS membranes [45].

Taken together, it will be very interesting to validate our findings by invitro measurements, for which different strategies might be proposed like NMR measurements of dimerizing GpA mutants with disturbed GxxxG motif (as in [42,45]) or of wild type GpA in thicker membranes (as in [45]). The best candidate for such an alternative interface is represented by cluster 81, which is the most stable cluster in long self-assemblies and which is also found after adaptation of the parameter for the definition of the helix conformation. The most important amino acids of this interface are I77, F78, M81 and A82 and so the investigation of the dimerization behavior of mutants disturbing both (the GxxxG motif and the interface of cluster 81) is an interesting experiment.

Remarkably, the occurrence of the different interfaces, their retention time and their transitions (Figure 1, Figure 2, Figure 3 and Figure 4) are strongly fatty acid chain dependent. For example the retention time of the most frequent interface (cluster 81) from long self-assembly simulations is highest in DPPC (Figure 3). This finding is in agreement with the observation that the Potential of Mean Force (PMF) for GpA association is highest in intermediately thick bilayers like DPPC and lower in thicker (DOPC) and thinner (DMPC) bilayers [46]. Thus, the retention times are a good measure for the stability of the corresponding interface. This in turn reveals that the NMR interface is more stable in thick bilayers concluded from highest observed retention times (Figure 4). Worth mentioning, the results obtained by long scale simulations (Figure 4) and by atomistic simulations for 1.5 ns [47] are comparable with respect to e.g., helix tilt or crossing angle, which documents that properties of transmembrane helices mainly depend on the bilayer thickness.

Flanking amino acids provide a second contribution for the dimer stability and formation [48,49]. Indeed, this notion is consistent with the enhanced retention time for interfaces of the 40 mer in comparison to the 25 mer (Figure 4). Although it is known that the LIxxGxxxGxxxT motif of the GpA helix is sufficient for association in *E.* *coli* membranes [50,51], the flanking amino acids most likely modulate the dynamics of the assembly *in vivo* as well.

Thus, we conclude that the interface formation of GpA depends on the surrounding fatty acids, the amino acids flanking the transmembrane region and the helix conformation. In turn, it is important to reproduce a native environment, either in biological or in in silico experiments, to get information on the native behavior of proteins. This holds true especially for mechanisms which relay on the hydrophobic thickness of membranes like the ratio and transition between left- and right-handed interface e.g., of EphA2 [44] or EpHA1 [43]. Translating our findings for the different membranes into a biological context suggests that modulation of dimerization interfaces of transmembrane proteins can be achieved by changes of the lipid composition, which is a common stresses reaction [52,53,54]. Such dependence of protein action and membrane dynamics has for example been described for Hik33 [55,56,57] or DesK [58]. Furthermore, there are several reports that the transport or enzyme activity of proteins depends on the bilayer thickness or curvature (e.g., reviewed in [59]). Remarkably, such lipid dependence has been reported for the hexose transporter band 4.5 [60], which is alike GpA localized in erythrocyte membranes. Thus, it is not unlikely that the function of GpA is modulated by membrane environment changes as well.

## 4. Methods

### 4.1. Simulation Details

Three different versions of the MARTINI force field were used to describe the protein structure: (i) the parameters used in Sengupta and Marrink 2010 (referred to as GpA2010; [26]); (ii) Martini2.1 [28]; (iii) Martini2.2 [29] parameters as created by the martinize.py script. The PC lipids with different fatty acids are standard MARTINI lipids [27]. All simulations were performed using GROMACS v4.5.5 [61] with standard MARTINI parameters: the neighbor lists are updated every 10th time step and a cutoff of 1.2 nm for the non-bonded interactions is applied (smooth switching from 0.0 to 1.2 nm for Coulomb potential and 0.9 to 1.2 nm for Lennard-Jones potential). A relative dielectric constant of 15 is used. For temperature (*T* = 320 K) and pressure coupling (semi-isotrope, *p* = 1 bar) the Berendsen thermostat and barostat are used [62]. All simulation times reported are raw simulation times and not multiplied by a special factor to consider the speed-up from coarse graining as used in other reports [63].

### 4.2. System Composition

The NMR structure (pdb:1afo—40 mer; [21]) was converted into a coarse grain model. The structure was truncated for simulations of the 25 amino acid long transmembrane helix (Glu72 to Arg96). For the self-assembly simulations helix A of the NMR structure was used. The stability of the dimer interface was tested by inserting the coarse grain structure based on NMR data [21], into a pre-equilibrated bilayer (protein:lipid:water ratio of 2:200:3000 (25 mer)/4500(40 mer)), followed by energy minimization and an MD run where the GpA dimer is position-restrained with a force constant of 1000 kJ·mol^−1^·nm^−2^. For the self-assembly the two single helices were inserted with a maximum distance from each other into a pre-equilibrated bilayer (protein:lipid:water ratio of 2:200:3000). The *N-* and *C*-terminus of the protein are uncharged and the simulation box has no net charge.

### 4.3. Details for the Short Self-Assembly Simulations

For short self-assembly simulations each inserted helix is rotated randomly around the helix axis to ensure that dimer formation is not influenced by the starting position. Each system is simulated for 0.3 µs and if no stable dimer is observed in the last frame continued for 0.1 µs. A dimer is considered as stable when at least 19 contacts occur between different amino acids of the two helices for a time span of 100 ns. 19 contacts is a suitable cutoff, because this number of contacts was derived from the simulation of the NMR dimer with the Martini2.2 force field (Figure S3). For the cluster analysis we used only the last frame of the short self-assembly trajectories.

### 4.4. Description of the Similarity to the NMR Structure and Clustering of Results

A contact map for each frame was calculated using the amino acids of the transmembrane part (25 mer). In the according figures, the *X*-axis indicates the residues of helix A and the *Y*-axis the residues of helix B. If residue *i* of helix A and residue *j* of helix B are in contact with each other with a distance shorter than 6 Å the value for the position (*i*,*j*) is set to one. If the distance is ≥6 Å, zero is assigned to (*i*,*j*). Distances are calculated with Yasara [64]. In the plot, the frequency of counts <6 Å are given in grey scale.

For the comparison to the NMR structure, the contact map of each frame is compared to the map of the NMR dimer and a simple score representing the similarity is defined:

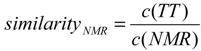
(1)
with *c*(*TT*) representing the number of contacts coinciding between NMR and MD structure, and with *c*(*NMR*) representing the total number of contacts between amino acids of the different helices of the NMR structure. In the transmembrane part of the NMR structure of GpA we observe 21 contacts (*c*(*NMR*) = 21).

For the clustering of all structures from the different self-assembly trajectories structures obtained every 50 ns were used. The contact maps modified to represent Boolean vectors are the basis for clustering. All frames that contain structures with less than 19 contacts were excluded from the clustering. Average linkage clustering with a cutoff of 0.5 for the cluster formation was used as implemented in SciPy [65]. A distance matrix for all contact maps is constructed using the dice dissimilarity [66] as implemented in SciPy [65].

### 4.5. Autocorrelation, Correlation and Fitting

For the (auto-) correlation plots we used Pearson’s correlation coefficient (*r*):

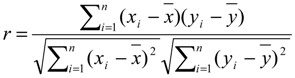
(2)
where is the number of randomly drawn simulations without replacement, and are frequencies of cluster *i* and are the averages. For testing the autocorrelation for *n* = 150, the first 150 interfaces were used for the distribution *X* and the remaining 150 interfaces for the distribution *Y*. In total the input orders were permuted 1000 times.

Afterwards a double exponential function is fitted onto the average correlation values using the least-squares fitting as implemented in SciPy [65]. As target function we use where *y* is the observed value, is the standard deviation of *y* and is the calculated value with . The parameters *a*, *b*, *c* and *d* are estimated from the data and afterwards optimized using least-squares fitting (Table 1).

### 4.6. Construction of the Transition Net

The transition net is built from the long self-assembly simulations. Only clusters with a frequency, which at least corresponds to the NMR cluster, are considered for the network (clusters 12, 14, 16, 42, 43, 44, 52, 63, 76 and 81). Each cluster forms a node, which is connected via an edge if a direct or indirect transition between the two clusters is observed in the trajectories. A transition is regarded as indirect if two clusters are connected via other clusters, which are not explicitly represented in the net. Shown are the three most frequent transitions for each cluster. Transitions occurring only once are not considered, because they are not reproduced in our data.

### 4.7. Backmapping and Comparison to NOE’s

The back mapping of the coarse grain structures to fine grain structures was performed using the back mapping tool BACKWARD [67]. In short, we used the charmm36 force field and did some energy minimizations and MD simulations with constant pressure using different time steps. The resulting structures in full atomistic resolution were compared to the maximal intermolecular distances obtained from the NMR experiments.

## 5. Conclusions

The self-assembly of a transmembrane segment depends on the helix length, the conformation of the helix and on the nature of the surrounding fatty acids. The latter influence the hydrophobic thickness and the fluidity of the membrane. By this property the nature of the fatty acid determines the interface formed shortly after assembly, the transitions between interfaces after dimer formation and the interface stability. The structure of the dimer interface depends to a certain extent on the force field parameter, because application of an elastic network was found to enhance the detection of the interface established by NMR experiments. Taken together, our results document on the one hand the importance of further force field development of protein parameters for the analysis of membrane domain assembly and on the other hand the requirement for correct modeling of membranes to obtain detailed information on the behavior of proteins in their native environment.

## Supplementary Files

Supplementary File 1
