# Supplementary material for: The Influence of Fatty Acids on the GpA Dimer Interface by Coarse-Grained Molecular Dynamics Simulation
Source: Int J Mol Sci. 2014 Aug 15;15(8):14247–68. doi: 10.3390/ijms150814247 (PMC4159849; doi:10.3390/ijms150814247)

Supplementary Information

Table S1. The 5 minimal distances between beads from different monomers of homodimeric bitopic α-helices with known NMR structure, which was converted into a CG representation for the analysis.

2ka2			2k9y			1afo		
**helix A**	**helix B**	**distance**	**helix A**	**helix B**	**distance**	**helix A**	**helix B**	**distance**
BBPHE	BBSER	3.10488	BBLEU	BBALA	3.2457	BBGLU	BBPRO	2.74635
BBPHE	BBSER	3.18935	BBLEU	BBALA	3.25337	BBILE	BBLYS	2.86863
BBLEU	BBPRO	3.2447	BBPHE	BBILE	3.25935	BBGLU	BBPRO	3.29006
SC1SER	SC2HIS	3.31513	BBPHE	BBILE	3.27329	BBILE	BBLYS	3.37511
BBGLY	SC1SER	3.31833	SC1SER	SC1LEU	3.56146	BBGLU	SC1PRO	3.49815
**2k1k**			**2ka1**			**2l2t**		
**helix A**	**helix B**	**distance**	**helix A**	**helix B**	**distance**	**helix A**	**helix B**	**distance**
BBLEU	BBTHR	3.64333	BBPHE	BBSER	3.18135	BBARG	BBLYS	3.15629
BBLEU	BBTHR	3.64489	BBLEU	BBPRO	3.2233	BBARG	BBLYS	3.16398
BBARG	BBSER	3.67019	BBLEU	BBPRO	3.25696	BBARG	BBTHR	3.6572
BBARG	BBSER	3.67661	BBPHE	BBSER	3.31495	BBARG	BBTHR	3.72581
BBVAL	BBARG	4.04698	BBARG	BBLEU	3.35745	SC3HIS	SC1GLN	3.78445
**2l9u**			**2loh**			**2l6w**		
**helix A**	**helix B**	**distance**	**helix A**	**helix B**	**distance**	**helix A**	**helix B**	**distance**
SC3PHE	SC3PHE	3.13432	BBSER	BBGLN	3.08917	BBGLN	BBLYS	3.12325
BBTHR	BBHIS	3.20186	BBASN	BBLYS	3.12417	BBGLN	BBLYS	3.28172
BBTHR	BBHIS	3.21512	BBVAL	BBGLY	3.3569	BBPRO	BBPHE	3.49944
SC2PHE	SC2PHE	4.06314	BBGLY	BBSER	3.3671	BBPRO	BBPHE	3.54458
SC3PHE	SC2PHE	4.11281	BBVAL	BBGLY	3.3774	BBGLN	SC1LYS	4.2117
**2j5d**			**2l34**					
**helix A**	**helix B**	**distance**	**helix A**	**helix B**	**distance**			
BBPHE	BBLEU	3.19401	BBARG	BBLEU	2.67973			
BBPHE	BBSER	3.32284	BBARG	BBLEU	2.71681			
BBLEU	BBTHR	3.37156	BBLEU	BBGLY	3.22939			
SC1SER	SC2HIS	3.38521	BBSER	BBPRO	3.40705			
BBLEU	BBTHR	3.45504	BBSER	SC1PRO	3.52303			

Figure S1. Representative Structures for the most frequent clusters. Shown are the contact plot for the whole cluster (as shown in Figure 1), the contact plot of the representative structure and the CG-structure. In the CG-structure all amino acids which are typical interface for the interface are colored in red, other residues of molecule A/molecule B are colored in light grey/dark grey. All backbone beads are show as small ball and side chain beads are shown as bigger balls. For celerity the distance between the Glycine residues of the GxxxG motif is indicated for the NMR like structures.

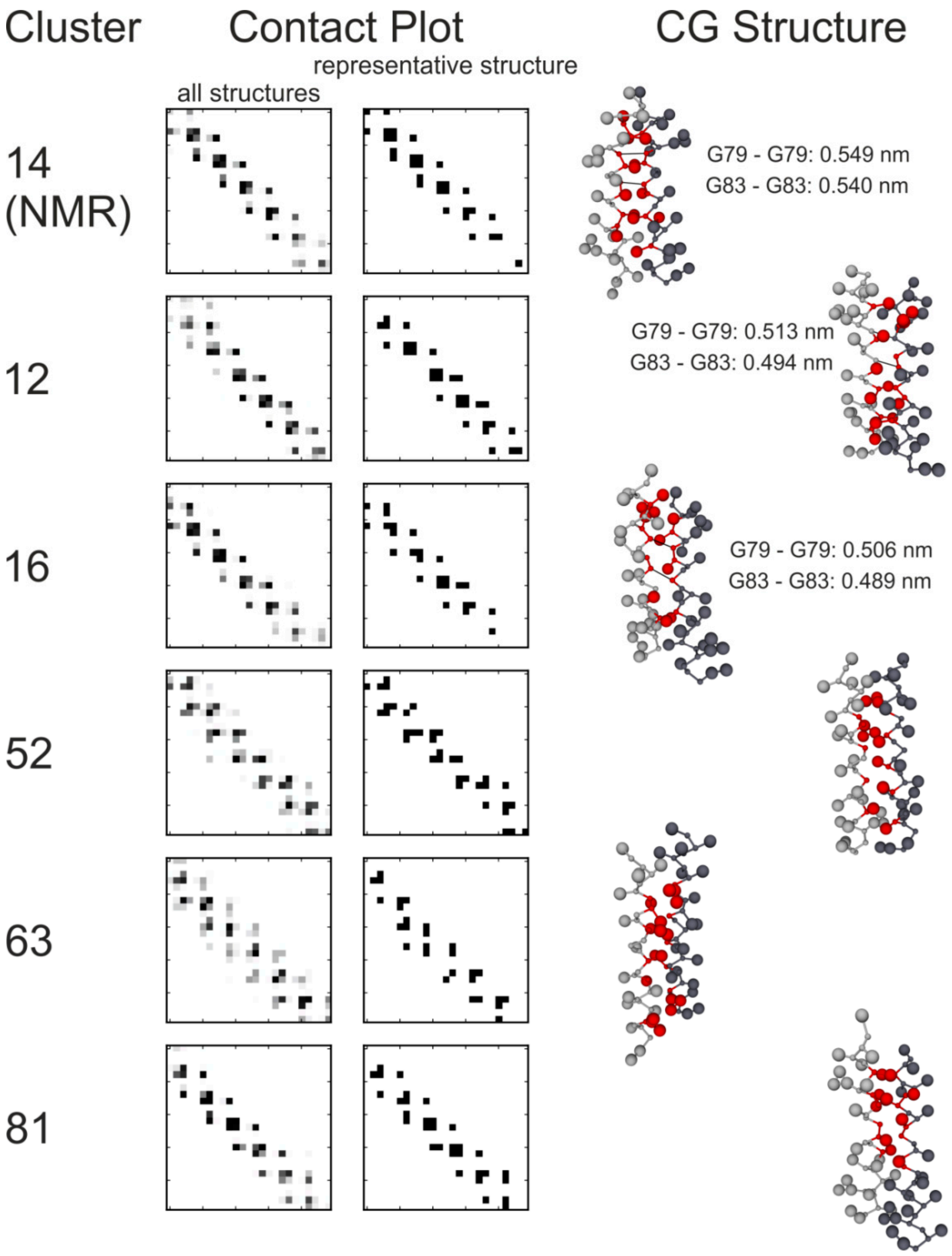

Figure S2. Frequency of Transitions between frequent clusters. Each of the eleven plots displays the transitions from one cluster to all other frequent clusters listed on the *X*-axis for the three different lipids (*Y*-axis). The darker the color the more frequent the transition from cluster x to y.

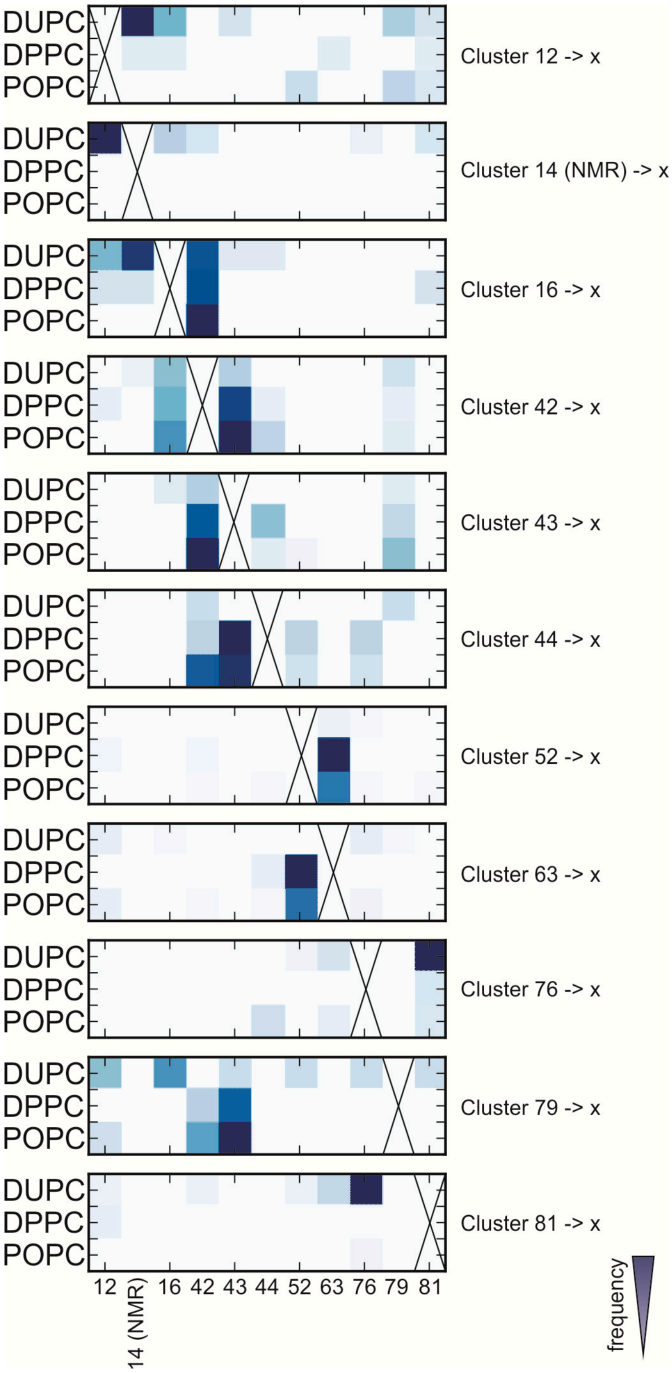

Figure S3. Number of contacts between amino acids. Number of contacts between amino acids of different helices (*X*-axis) is plotted against the frequency (*Y*-axis) observed in the simulations of the GpA_TM NMR structure (25 mer) with the Martini 2.2 force field and different fatty acids. The red, dashed line corresponds to the cutoff used for the short self-assembly simulations: The mean of observed contacts per frame is 31.79 and the standard derivation 4.01 ($\lfloor \text{mean} + 3\sigma \rfloor = \lfloor 19.76 \rfloor = 19$).

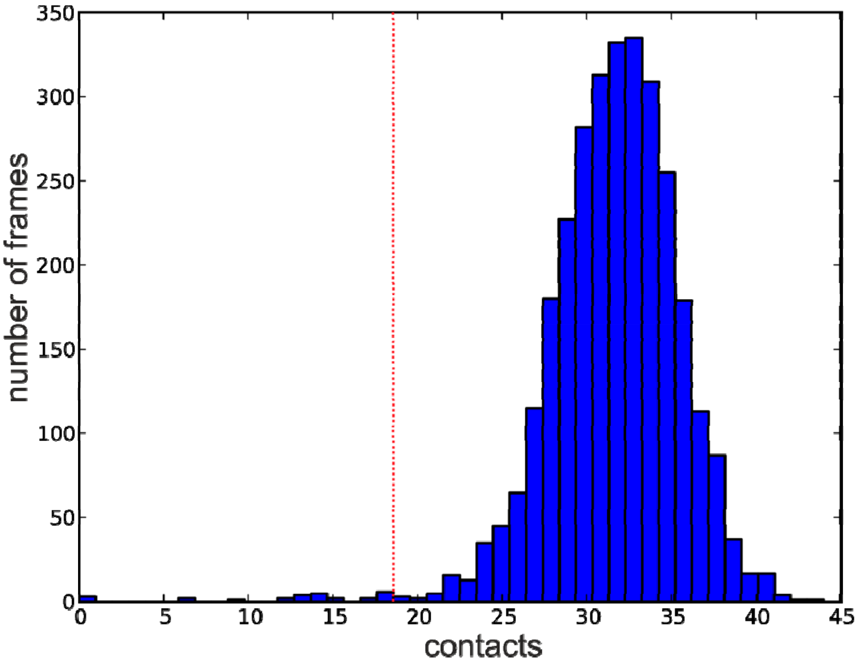

Supplement: Supplementary File 1 [file ijms-15-14247-s001.pdf]
